# Signal Transmission of Biological Reaction-Diffusion System by Using Synchronization

**DOI:** 10.3389/fncom.2017.00092

**Published:** 2017-10-10

**Authors:** Lingli Zhou, Jianwei Shen

**Affiliations:** ^1^School of Mathematics and Statistics, Zhengzhou University, Zhengzhou, China; ^2^Institute of Applied Mathematics, Xuchang University, Xuchang, China

**Keywords:** random walk, signal transmission, synchronization, reaction-diffusion system, diffusion coupling, structure adaptation

## Abstract

Molecular signal transmission in cell is very crucial for information exchange. How to understand its transmission mechanism has attracted many researchers. In this paper, we prove that signal transmission problem between neural tumor molecules and drug molecules can be achieved by synchronous control. To achieve our purpose, we derive the Fokker-Plank equation by using the Langevin equation and theory of random walk, this is a model which can express the concentration change of neural tumor molecules. Second, according to the biological character that vesicles in cell can be combined with cell membrane to release the cargo which plays a role of signal transmission, we preliminarily analyzed the mechanism of tumor-drug molecular interaction. Third, we propose the view of synchronous control which means the process of vesicle docking with their target membrane is a synchronization process, and we can achieve the precise treatment of disease by using synchronous control. We believe this synchronous control mechanism is reasonable and two examples are given to illustrate the correctness of our results obtained in this paper.

## 1. Introduction

In recent years, many scientists attempt to understand the mechanism behind the biological phenomena and how it works. For instance, Maini et al. (1997, 2012) studied the biological pattern formation in reaction diffusion theory. Hung et al. (2016) introduced the effect of MicroRNA for zebrafish larvaes' cold shock in the view of gene regulation. Stepicheva and Song (2016) showed that miR-31 regulates diverse cellular and developmental processes by targeting genes involved in cell proliferation, apoptosis, cell differentiation, and cell motility. Brophy and Voigt (2016) built a synthetic system in *Escherichia coli* to study how antisense transcription can change the expression of a gene, and determined the relative contributions of antisense RNA and transcriptional interference to repressing gene expression and introduce a biophysical model to capture the impact of RNA polymerase collisions on gene repression.

It's well known that there is a signal transmission in the biological system at all times, the correct biochemical reaction can not be separated from these signal transmission. Thompson and Holbrook (2004) used a previously developed dimensionless model of phloem transport to demonstrate the mechanism behind the sieve tube's capacity to rapidly transmit pressure or the magnitude and axial gradient of apoplastic water potential. Faria et al. (2014) propose a model of an intra-cellular transmission system of genetic information to identify a mathematical structure in DNA sequences where such sequences are biologically relevant, the characterization of this model may contribute to the development of a methodology that can be applied in mutational analysis and polymorphisms, production of new drugs and genetic improvement. There are many papers discover the drug molecules' transmission effect (Khuda-Bukhsh, 2003; Slowing et al., 2011; Viernes et al., 2014). However, there is little attention paid to the precise treatment of cancer, such as nerve-tumor. Although it is very challenging to carry out precise treatment of tumor molecules in the nervous system, it has greatly improved the efficiency of tumor treatment. Precision therapy refers to the combination of drug molecules and cancer causing sites, which can cause tumor cells to die specifically. Drug molecules are transported in the body in the form of vesicles wrapped, then the precision transport of vesicles is also a powerful certification for the precise treatment of tumor molecules. In 2013, the Nobel prize in physiology or medicine was awarded to three scientists who revealed the secrets of how cells organize their transport systems, that's to say some molecules which wrapped in vesicles how to be delivered to the correct cell target at the right time. Vesicle trafficking is an important component of the material transport mechanism of cells, it can deliver the right cellular cargo to the right destination at the right time (such as insulin, neurotransmitter release). The essence of cellular logistics is the precise transfer and delivery of goods. The process of precise transport is just like the refueling of a flying aircraft by an aerial tanker. When it can refuel, we also call the two aircrafts reached a state of synchronization. Inspired by this example, we want to explain the mechanism behind the precise treatment of tumor molecules from the view of synchronization. For the movement of tumor molecules, biologists believe that cancer cells follow a random walk model in the two-dimensional plane (Wu et al., 2014). So in this work, we try to build the model from the view of real world by using Brownian motion, and our problem is how to explain the precise therapeutic mechanism of tumor molecules through mathematical models. Our work is to realize the synchronization between them, then the two can interact: the drug molecules destroy the tumor cells, thereby preventing normal cell cancerization. It's like an air refuelling tankers which has being flying to the target location, is about to start fueling. So the state of sync also represents an effective treatment.

Motivated by the discussion above, this paper aims to realize the precise control of neural tumor molecules by drug molecules which can be modeled as a process of synchronization for a class of partial differential systems. To this end, we designed two controllers which contains feedback controller, structure adaptive controller (Zeng-Rong and Ji-Gui, 2006) and diffusion controller (Wu and Chen, 2012). The main structure of this paper are as follows:In section 2, we give the mathematical models for the neural tumor molecules and the drug molecules respectively, some necessary assumptions and the definition of structure adaptation are also given in this section. In section 3, complete synchronization for the proposed model by using two controllers will be studied. Then, in section 4, numerical simulation is presented to show the effectiveness of the theoretical results. Finally, section 5 provides some conclusions and future research topics.

## 2. The model

**Figure 1:**
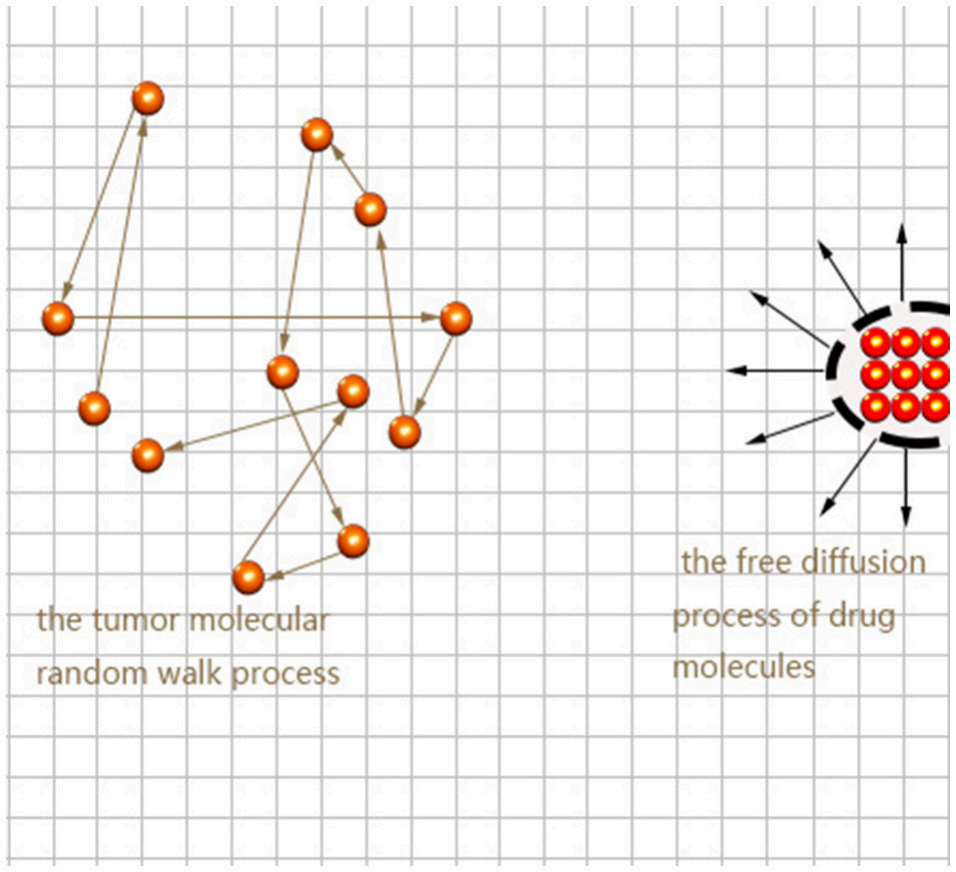
Movement path of tumor molecules and drug molecules.

Here we consider the interaction between neural tumor molecules and drug molecules in the plane, which is a random walk process and a free diffusion process respectively. Schematic diagram is shown in Figure 1.

We use the following langevin equation to describe the trajectory of neural tumor molecules in the bounded plane. In general, it can be written as:

(2.1) $$\begin{array}{lll} \frac{dx}{dt} & = & f_{1}\left(x,y\right)+\xi_{1}\left(t\right)\\ \frac{dy}{dt} & = & f_{2}\left(x,y\right)+\xi_{2}\left(t\right) \end{array}$$

where (*x, y*) is the coordinates of neural tumor molecules, *f*_*i*_(*x, y*) is the viscous resistance from fluid such as cellular fluid. In order to simplify the model, we choose a simple linear representation to *f*_*i*_(*x, y*), that's to say: *f*_1_(*x, y*) = −*k*_1_*x, f*_2_(*x, y*) = −*k*_2_*y*. ξ_*i*_(*t*) is Gauss white noise which satisfies: $\langle \xi_{i}\left(t\right)\rangle =0,\langle \xi_{i}\left(t\right)\xi_{i}\left(t^{\prime }\right)\rangle =D_{i}\delta \left(t-t^{\prime }\right)$. When the initial value (*x*_0_, *y*_0_) is given, for each sample function ξ_*i*_(*t*), the system (Equation 2.1) has the only determinate solution. Because the values of ξ_*i*_(*t*) at different time is random and independent, the trajectory of neural tumor molecules is a Markov process. Gao (2010) has introduced how to translate Langevin equation to Fokker-Planck equation in one-dimensional space, we can derive the following Fokker-Planck equation corresponding to Equation (2.1) in two-dimensional space:

(2.2) $$\begin{array}{lll} \frac{\partial P\left(x,y,t\right)}{\partial t} & = & -\frac{\partial }{\partial x}\left(f_{1}\left(x,y\right)P\right)\;-\;\frac{\partial }{\partial y}\left(f_{2}\left(x,y\right)P\right)\\ +\;\frac{D_{1}}{2}\frac{\partial^{2}}{\partial x^{2}}P\;+\;\frac{D_{2}}{2}\frac{\partial^{2}}{\partial y^{2}}P \end{array}$$

where *P*(*x, y, t*) is the probability density of neural tumor molecules in the cell. If we assumpted the two noise intensity *D*_1_, *D*_2_ in Equation (2.1) are equal to *D*, two viscous resistance coefficients are equal: *k*_1_ = *k*_2_ = *K*, then Equation (2.2) can be written in the alternate form:

(2.3) $$\begin{array}{l} \frac{\partial P\left(x,y,t\right)}{\partial t}=f\left(P\right)+\frac{D}{2}\Delta P \end{array}$$

where $f\left(P\right)=2KP+K\left(x\frac{\partial P}{\partial x}+y\frac{\partial P}{\partial y}\right)$.

On the other hand, we use the reaction diffusion equation to express the concentration of drug molecules, it can be written as:

(2.4) $$\begin{array}{l} \frac{\partial Q\left(x,y,t\right)}{\partial t}=-hQ+D_{2}\Delta Q. \end{array}$$

where *h, D*_2_ are the absorption coefficient and diffusion coefficient, respectively. When *t* = 0, *Q*(*x, y*) approximates to the point source pulse function at injection point, so *Q*(*x, y*, 0) = *Lδ*(*x, y*), and *L* represents injection volume.

Up to now, we have get the concentration equations of neural tumor molecules and drug molecules respectively, i.e., Equations (2.3) and (2.4). Our next work is to disscuss the signal transmission between the two from the view of synchronization.

## 3. Realization of complete synchronization and control term

**Figure 2:**
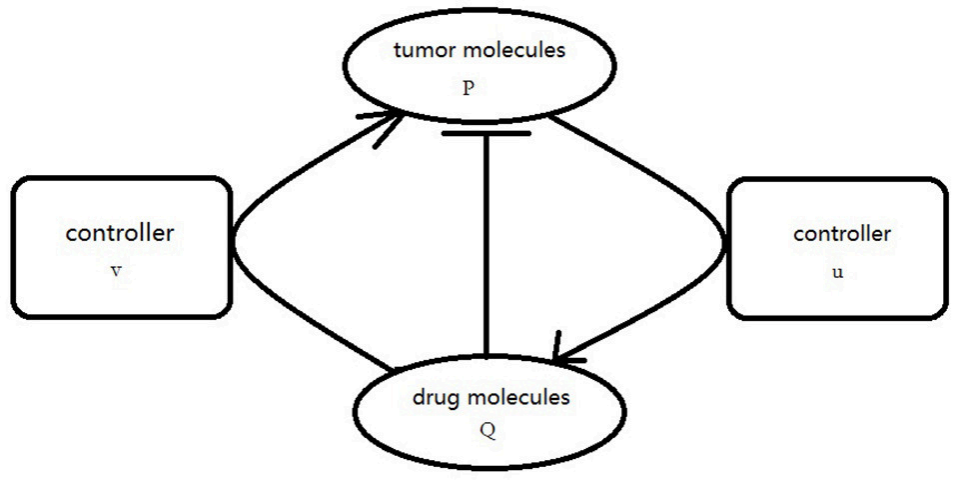
Schematic diagram of coupling effect between neural tumor molecules and drug molecules.

Firstly, we give the general description of CS betweenn two different reaction diffusion systems. Consider the following system:

(3.5) $$\begin{array}{l} \begin{array}{c} \frac{\partial P\left(x,y,t\right)}{\partial t}=f\left(P\right)+D_{1}\Delta P\\ \frac{\partial Q\left(x,y,t\right)}{\partial t}=g\left(Q\right)+D_{2}\Delta Q \end{array} \end{array}$$

where *f* is defined as Equation (2.3), *g*(*Q*) = −*hQ*, $D_{1}=\frac{D}{2}$. Our goal is to choose the appropriate controllers for (3.5) so that the orbit of the *P* component evevtually synchronized to the orbit of *Q* component. In order to realize the CS, the proper coupling term *v*(*P, Q*), *u*(*P, Q*) is add to the *P* component and *Q* component of Equation (3.5) as usual. The simplified graph is shown in Figure 2.

So the coupling system is expressed as:

(3.6) $$\begin{array}{l} \begin{array}{cc} \frac{\partial P\left(x,y,t\right)}{\partial t} & =f\left(P\right)+D_{1}\Delta P+v\left(P,Q\right)\\ \frac{\partial Q\left(x,y,t\right)}{\partial t} & =g\left(Q\right)+D_{2}\Delta Q+u\left(P,Q\right) \end{array} \end{array}$$

such that $\underset{t\to \infty }{\lim}|P\left(x,y,t\right)-Q\left(x,y,t\right)|=0$.

Now we propose that the two control terms in Equation (3.6) can be expressed in the following form:

(3.7) $$\begin{array}{l} \begin{array}{c} v\left(P,Q\right)=v_{1}\left(P,Q\right)+v_{2}\left(P,Q\right)\\ u\left(P,Q\right)=u_{1}\left(P,Q\right)+u_{2}\left(P,Q\right) \end{array} \end{array}$$

where *v*_1_(*P, Q*) is the feedback part in the control term, *u*_1_(*P, Q*) reflects the structure adjustment in the control term and *v*_2_(*P, Q*), *u*_2_(*P, Q*) express the diffusion coupling in the control term. They are continuous functions that can be taken as:

(3.8) $$\begin{array}{l} \begin{array}{cc} v_{1}\left(P,Q\right) & =\varepsilon_{1}\left(P-Q\right)\\ v_{2}\left(P,Q\right) & =\left(\varepsilon_{2}d-D_{1}\right)\Delta Q\\ u_{1}\left(P,Q\right) & =\varepsilon_{2}\left[f\left(P\right)-g\left(P\right)\right]\\ u_{2}\left(P,Q\right) & =\left(\varepsilon_{2}d-D_{2}\right)\Delta P \end{array} \end{array}$$

where *d* = *D*_1_ + *D*_2_.

Let *e* = *Q* − *P*, the error evolution equation of Equation (3.6) reads:

(3.9) $$\begin{array}{l} \begin{array}{cc} ė & =g\left(Q\right)-f\left(P\right)+D_{2}\Delta Q-D_{1}\Delta P+\varepsilon_{1}e+\varepsilon_{2}\left[f\left(P\right)-g\left(P\right)\right]\\ +\left(D_{1}-\varepsilon_{2}d\right)\Delta Q-\left(D_{2}-\varepsilon_{2}d\right)\Delta P\\ =\left[g\left(Q\right)-g\left(P\right)\right]+\varepsilon_{1}e+\left(1-\varepsilon_{2}\right)\left[g\left(P\right)-f\left(P\right)+d\Delta e\right]. \end{array} \end{array}$$

For system (Equation 3.9), we give three general assumptions as follows:

(H1) For any *m, n* ∈ Ω ∈ *R*, there exists a constant *l* > 0 satisfying

$$\begin{array}{l} \left|g\left(m\right)-g\left(n\right)|\leq l|m-n\right| \end{array}$$

Where Ω is a bounded set. This condition is called the uniform Lipschitz condition.

(H2) System (Equation 3.9) is eventually dissipative, namely there is a bounded set Ω_1_ × Ω_2_ ∈ *R* × *R* such that the orbit (*P*(*t*), *Q*(*t*)) starting from any initial point (*P*_0_, *Q*_0_) eventually enters Ω_1_ × Ω_2_. Thus, we can obtain that the functions *f* and *g* satisfy:

$$\begin{array}{l} |g\left(P\left(t\right)\right)-f\left(P\left(t\right)\right)|<M, \end{array}$$

for sufficient large *t* > 0.

(H3) The Laplasse operator is bounded. There exists a constant *N* > 0 such that Δ*P* < *N*, Δ*Q* < *N* for any *t* > 0.

Here the control strength ε_1_ and ε_2_ will be self-adapted according to the following update law:

(3.10) $$\begin{array}{l} \begin{array}{cc} \dot{\varepsilon_{1}} & =-e^{2}\\ \varepsilon_{2} & =1-\frac{e}{M+dN} \end{array} \end{array}$$

For the 4-system, consisting of the error equation (3.9) and self-adaptive equation (3.10), we introduce the following non-negative function:

$$\begin{array}{l} V=\frac{1}{2}e^{2}+\frac{1}{2}{\left(\varepsilon_{1}+L\right)}^{2}, \end{array}$$

Where *L* > *l* + 1 is a constant. By differentiating the function *V* along the trajectories, which enter Ω_1_ × Ω_2_ after a suffient large *t*, of the augmented system, we obtain

(3.11) $$\begin{array}{ll} \dot{V} & =eė+\left(\varepsilon_{1}+L\right)\dot{\varepsilon_{1}}\\ =e\left[\left(g\left(Q\right)-g\left(P\right)\right)+\varepsilon_{1}e+\left(1-\varepsilon_{2}\right)\left(g\left(P\right)-f\left(P\right)+d\Delta e\right)\right]\\ \;-\left(\varepsilon_{1}+L\right)e^{2}\\ =e\left[\left(g\left(Q\right)-g\left(P\right)\right)+\frac{e}{M+dN}\left(g\left(P\right)-f\left(P\right)+d\Delta e\right)\right]-Le^{2}\\ \leq \left(l+1-L\right)e^{2}\\ \leq 0 \end{array}$$

It's obvious that $\dot{V}=0$ if and only if *e* = 0. Then according to the invariance principle of differential equations, starting from arbitrary initial values of the augmented system, the orbit converges asymptotically, i.e., *Q* − *P* → 0, $\varepsilon_{1}\to {\tilde{\varepsilon }}_{1}$, ε_2_ → 1 as *t* → ∞. The CS between the two different reaction diffusion systems is achieved.

In the coupling function, *v*_1_(*P, Q*) represent the feedback part. Under the effect of this part, the distance between *P*(*x, y*) and *Q*(*x, y*) is gradually decreasing until converging to zero. On the other hand, *u*_1_(*P, Q*) plays a role of adjusting the structure of the *Q*-subsystem gradually. Its strength ε_2_ is controlled by *e*. When the distance between *P*(*x, y*) and *Q*(*x, y*) is decreasing gradually, the effect of structure adjustment strengthens. In addition, under the effect of the *v*_2_(*P, Q*), *u*_2_(*P, Q*), the distance between *P*(*x, y*) and *Q*(*x, y*) result from respective diffusion could be eliminated, eventually the CS fulfilled.

## 4. Numerical simulation

In this section, we give the numerical results for the coupling system of the previous section (Equation 3.6), that is:

(4.12) $$\begin{array}{ll} \frac{\partial P\left(x,y,t\right)}{\partial t} & =2KP+\varepsilon_{1}\left(P-Q\right)+K\left(x\frac{\partial P}{\partial x}+y\frac{\partial P}{\partial y}\right)\\ \;+D_{1}\Delta P+\left(\varepsilon_{2}d-D_{1}\right)\Delta Q\\ \frac{\partial Q\left(x,y,t\right)}{\partial t} & =-hQ+\varepsilon_{2}\left[\left(2K+h\right)P+K\left(x\frac{\partial P}{\partial x}+y\frac{\partial P}{\partial y}\right)\right]\\ \;+\left(\varepsilon_{2}d-D_{2}\right)\Delta P+D_{2}\Delta Q\\ \frac{\partial \varepsilon_{1}\left(x,y,t\right)}{\partial t} & =-{\left(P-Q\right)}^{2} \end{array}$$

where $\varepsilon_{2}=1-\frac{Q-P}{M+dN},d=D_{1}+D_{2}$, (*K, h, D*_1_, *D*_2_, *M, N*) are system parameters.

We assume that neural tumor molecules and drug molecules move in a bounded planar region, that is: (*x, y*) ∈ [0, 100] × [0, 100], and drug injection point is at (1, 1). Morever, because the *P*(*x, y, t*) in system (Equation 2.2) represents the probability density of neural cancer cells, we define *P* ∈ [0, 1], *Q* ∈ [0, 1] in this paper, and error *e* between them satisfied: *e* ∈ [−1, 1]. So we put the boundary conditions and initial conditions for system (Equation 4.12) as follows: *P*(*x, y*, 0) = *sin*(2π*x*)*cos*(2π*y*), *Q*(*x, y*, 0) = δ(1, 1), ε_1_(*x, y*, 0) = 0, *P*(0, *y, t*) = *P*(100, *y, t*) = *Q*(*x*, 0, *t*) = *Q*(*x*, 100, *t*) = 0, *Q*(0, *y, t*) = *Q*(100, *y, t*) = *Q*(*x*, 0, *t*) = *Q*(*x*, 100, *t*) = 0, ε_1_(0, *y, t*) = ε_1_(100, *y, t*) = ε_1_(*x*, 0, *t*) = ε_1_(*x*, 100, *t*) = 0.

**Figure 3:**
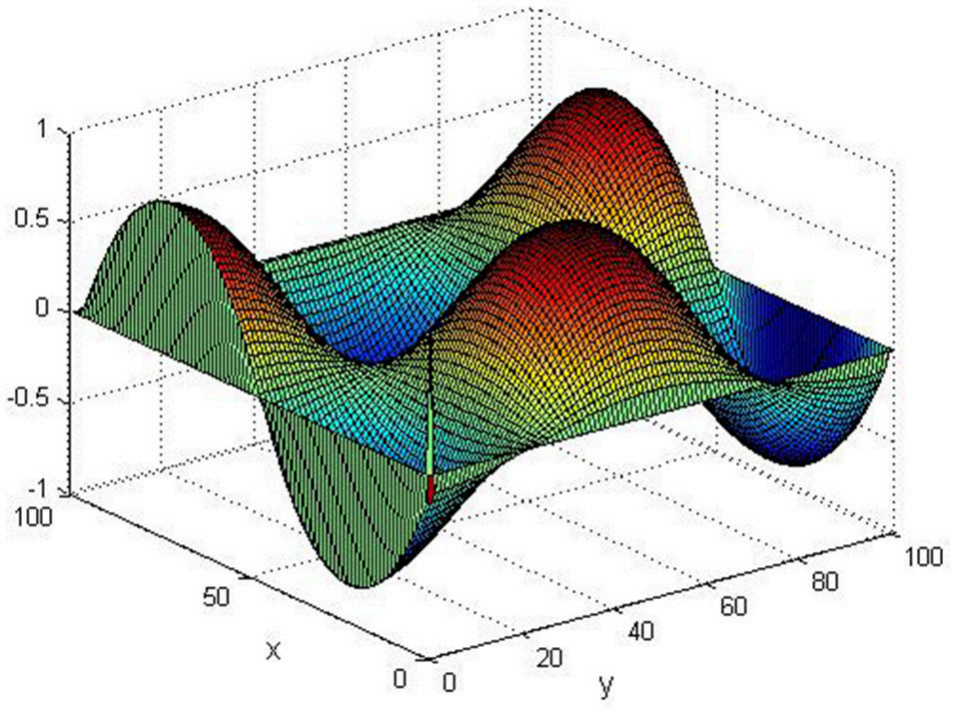
Simulation results of *e* at *t* = 0.

**Figure 4:**
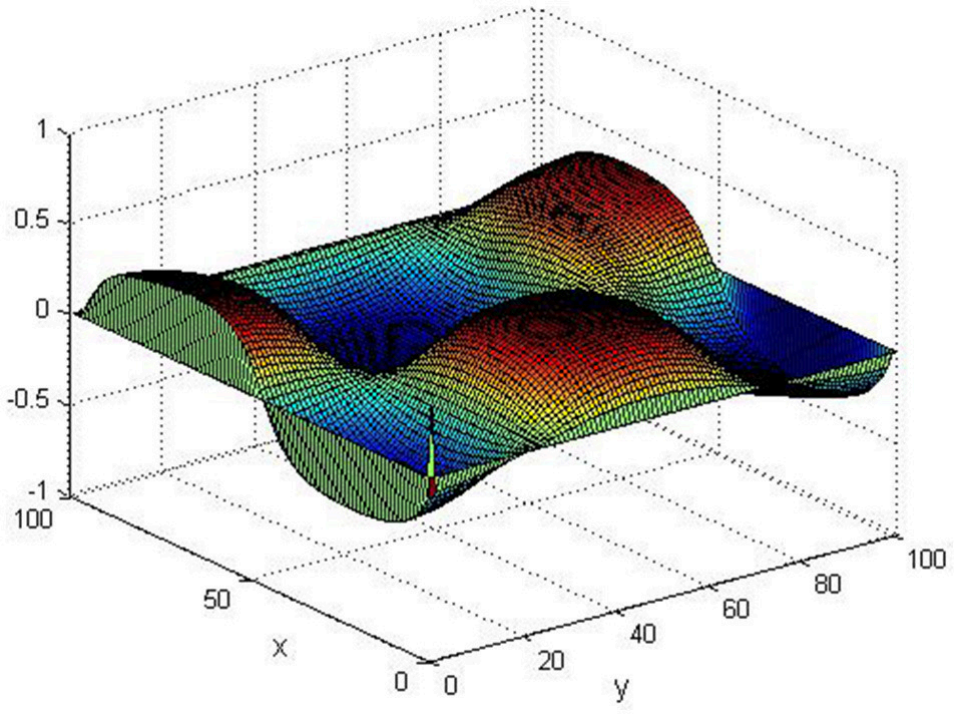
Simulation results of *e* at *t* = 2.

**Figure 5:**
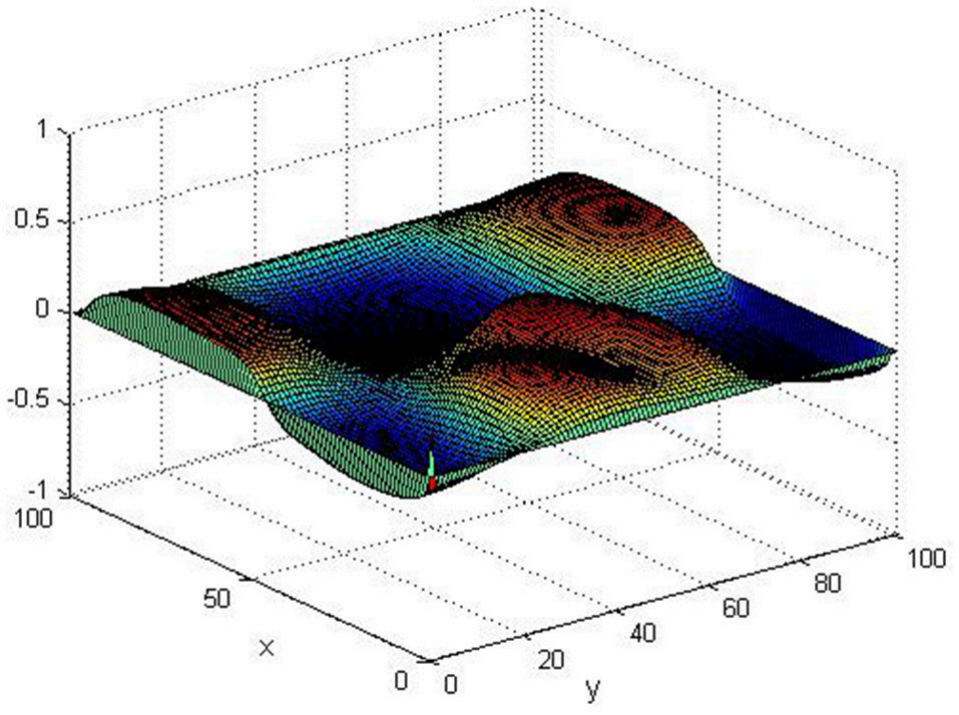
Simulation results of *e* at *t* = 5.

**Figure 6:**
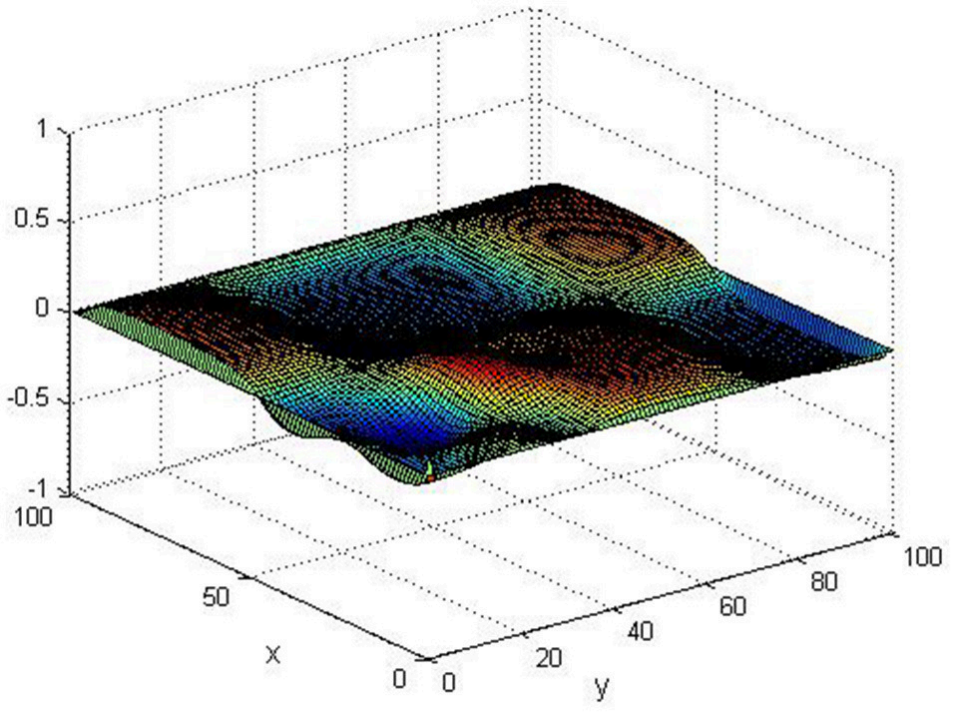
Simulation results of *e* at *t* = 10.

Take (*K, h, D*_1_, *D*_2_, *M, N*) = (0.1, 0.1, 0.01, 0.02, 200, 100), the state *e* = *Q*(*x, y, t*)−*P*(*x, y, t*) are shown in the Figures 3–6 at different times. We can read from these figures that the coupled systems are of asymptotical synchronization with the increase of time. In other words, the signal between the two is effectively transmitted.

**Figure 7:**
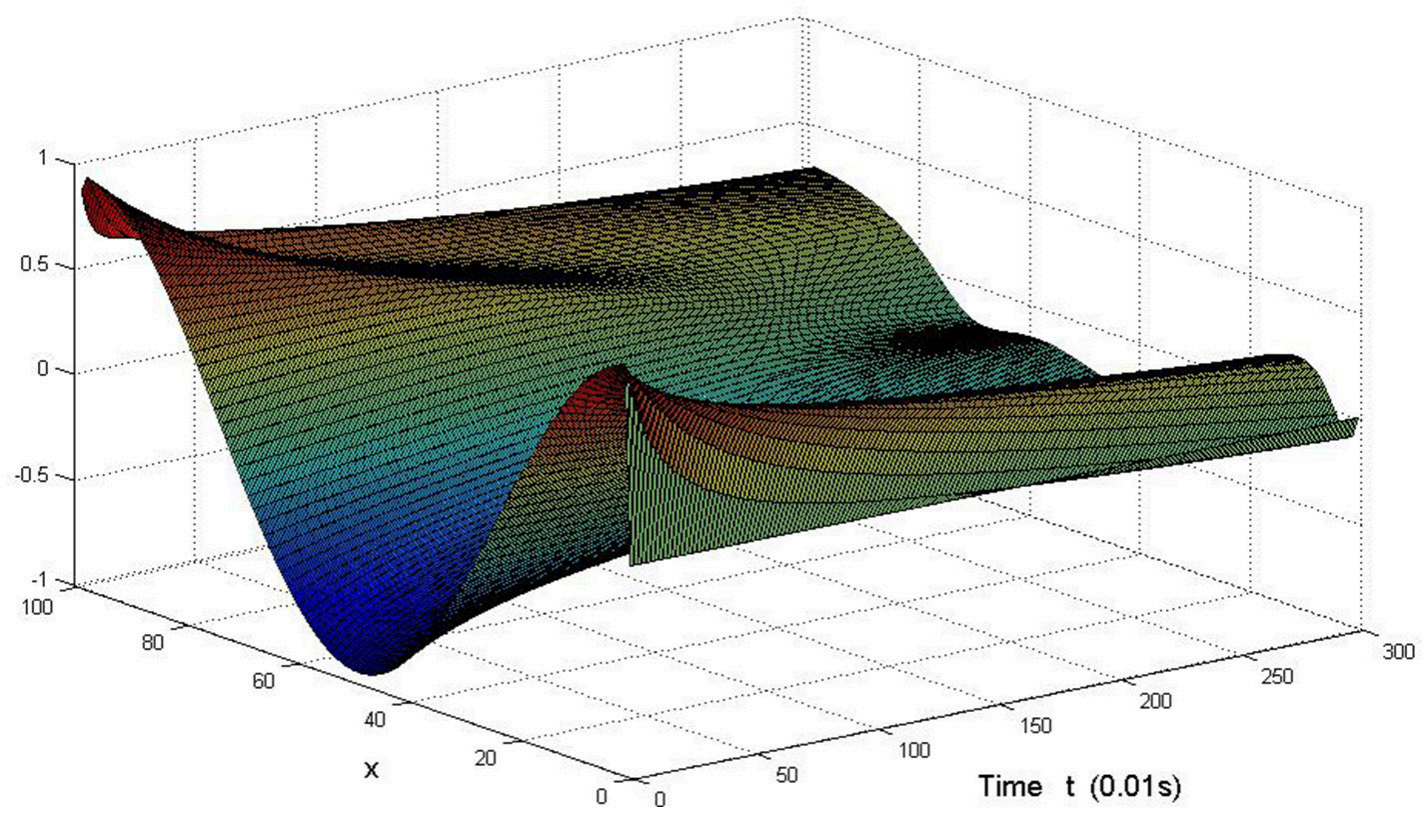
Difference between *P* and *Q* with the change of time at *y* = 20 in system (Equation 4.12).

**Figure 8:**
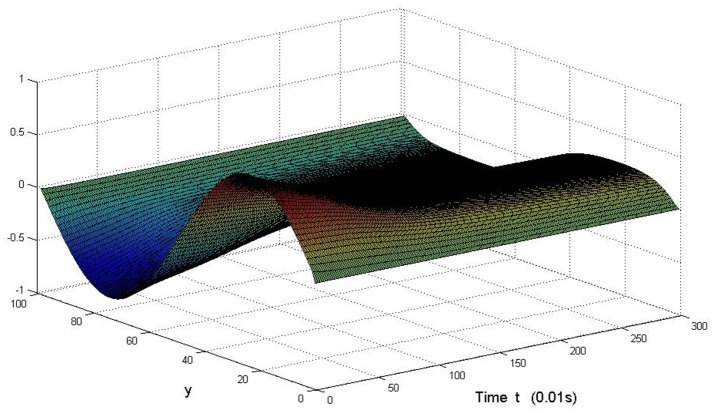
Difference between *P* and *Q* with the change of time at *x* = 90 in system (Equation 4.12).

Here we also provide figures to illustrate the difference between *P* and *Q* at some fixed space point. We take *y* = 20 from Figure 7, and *x* = 90 from Figure 8, we can also see the asympotical synchronization of the coupled systems (Equation 4.12). It also shows that it is reasonable for us to explain the mechanism of signal transduction in the view of synchronization.

## 5. Conclusion and future research issues

This paper considered the signal transmission between neural tumor molecules and drug molecules from the perspective of synchronization, we constructed the synchronization error dynamic, and turned the synchronization problems of coupled system to the stabilization problems of the synchronization error dynamic which can be analyzed via Lyapunov method. Realization of synchronization is also used to verify the effective transmission of the signal. So our paper is helpful to understand the signal transmission mechanism of biological reaction-diffusion system. In addition, the model has many parameters, and each parameter represents one or more biometric features, the biologist can relaize the control by adjusting the different parameter, and our work is of practical significance.

The characteristic of stochastic dynamics is a remarkable feature of biological systems. Because of the presence of noise, the various mechanisms of action in organisms often show great randomness. Study on the mechanism of these random effects from the master equation may be any other general type, and they can explain the mechanisms behind complex interaction more effectively. How to use the master equation to model the random moving objects is challenging, which is our next research topic.

## Author contributions

JS proposed the idea how to control the desease by using sychronization. LZ gave the proof of results and numerical simulation.

### Conflict of interest statement

The authors declare that the research was conducted in the absence of any commercial or financial relationships that could be construed as a potential conflict of interest.
